# Predicting Knee Joint Instability Using a Tibio-Femoral Statistical Shape Model

**DOI:** 10.3389/fbioe.2020.00253

**Published:** 2020-04-17

**Authors:** Pietro Cerveri, Antonella Belfatto, Alfonso Manzotti

**Affiliations:** ^1^Department of Electronics, Information and Bioengineering, Polytechnic University of Milan, Milan, Italy; ^2^Orthopaedic and Trauma Department, “Luigi Sacco” Hospital, ASST FBF-Sacco, Milan, Italy

**Keywords:** knee alignment, knee instability, femur shape, tibia shape, statistical shape model (SSM)

## Abstract

Statistical shape models (SSMs) are a well established computational technique to represent the morphological variability spread in a set of matching surfaces by means of compact descriptive quantities, traditionally called “modes of variation” (MoVs). SSMs of bony surfaces have been proposed in biomechanics and orthopedic clinics to investigate the relation between bone shape and joint biomechanics. In this work, an SSM of the tibio-femoral joint has been developed to elucidate the relation between MoVs and bone angular deformities causing knee instability. The SSM was built using 99 bony shapes (distal femur and proximal tibia surfaces obtained from segmented CT scans) of osteoarthritic patients. Hip-knee-ankle (HKA) angle, femoral varus-valgus (FVV) angle, internal-external femoral rotation (IER), tibial varus-valgus (TVV) angles, and tibial slope (TS) were available across the patient set. Discriminant analysis (DA) and logistic regression (LR) classifiers were adopted to underline specific MoVs accounting for knee instability. First, it was found that thirty-four MoVs were enough to describe 95% of the shape variability in the dataset. The most relevant MoVs were the one encoding the height of the femoral and tibial shafts (MoV #2) and the one representing variations of the axial section of the femoral shaft and its bending in the frontal plane (MoV #5). Second, using quadratic DA, the sensitivity results of the classification were very accurate, being all >0.85 (HKA: 0.96, FVV: 0.99, IER: 0.88, TVV: 1, TS: 0.87). The results of the LR classifier were mostly in agreement with DA, confirming statistical significance for MoV #2 (*p* = 0.02) in correspondence to IER and MoV #5 in correspondence to HKA (*p* = 0.0001), FVV (*p* = 0.001), and TS (*p* = 0.02). We can argue that the SSM successfully identified specific MoVs encoding ranges of alignment variability between distal femur and proximal tibia. This discloses the opportunity to use the SSM to predict potential misalignment in the knee for a new patient by processing the bone shapes, removing the need for measuring clinical landmarks as the rotation centers and mechanical axes.

## 1. Introduction

**Figure 1:**
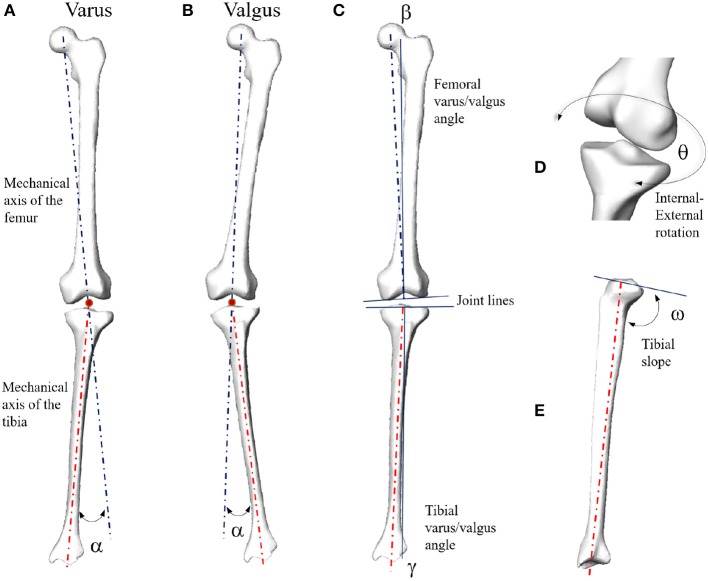
Mechanical angular deformity (α) of the knee joint in the frontal plane (**A**: varus; **B**: valgus). Femoral (β) and tibial (γ) varus/valgus angles computed with respect to the corresponding joint lines **(C)**. In case of parallel joint lines, β + γ = α. Internal-external rotation **(D)** and tibial slope **(E)**.

The three-dimensional (3D) rotation of the femur with respect to the tibia, called tibio-femoral alignment, is a fundamental clinical index in knee diagnosis and surgical planning, as it can be correlated to a large extent to the kinematic instability of the joint (Laxafoss et al., 2013; Thienpont et al., 2014). This 3D rotation is represented by five main angular variables, namely the hip-knee-ankle, femoral varus-valgus, and tibial varus-valgus angles, describing the knee stability in the frontal plane, and the internal-external femoral rotation and tibial slope, for the axial and sagittal alignments, respectively (Salenius and Vankka, 1975; Fitzpatrick et al., 2011; Schatka et al., 2018; Maillot et al., 2019). Specific bony landmarks (e.g., head center in the proximal femur, epicondyles and intercondylar fossa in the distal femur, epicondyles and frontal tuberosity in the proximal tibia, malleoli of the distal fibula, and the distal tibia) are mandatory for computing anatomical and mechanical axes and the corresponding tibio-femoral alignment in the knee (Lyras et al., 2016; Bennett et al., 2018). Clinical practice involves the manual detection of the landmarks on tomographic images or 3D reconstructed surfaces of bones and soft tissues. Both methods are time-consuming and prone to detection errors, even when performed by radiological and orthopedic experts. In order to improve landmark detection and tibio-femoral alignment computation, novel methodologies and tools, taking both semi- and fully-automatic approaches, have been proposed in the literature (De Momi et al., 2009; Cerveri et al., 2010; Subburaj et al., 2010; Kainz et al., 2015). However, such tools can fail in the case of large pathological deformations of the bony shapes. Indeed, as the degeneration progresses, the bony morphology deviates from the physiological shape, making the landmarks difficult to measure or even meaningless. In this scenario, landmark-free tools such as statistical shape models (SSMs) can represent an alternative for the evaluation of the knee joint alignment. SSMs have been extensively studied because of their ability to represent a set of matching surfaces synthetically in terms of a representative shape, namely the average surface of the set, and distinct morphological features, usually called “modes of variation” (MoVs). The magnitude of each MoV outlines the extent to which the morphological aspect it encodes is present in the set. Applications of bony surface SSMs in biomechanics and clinics have spanned anatomical and developmental studies (Li et al., 2010; Zhu and Li, 2011; Mutsvangwa et al., 2015; Baumbach et al., 2017; Wang and Shi, 2017; Zhang and Besier, 2017), shape anomaly staging (Van Haver et al., 2014; Agricola et al., 2015; Zhang et al., 2016; Cerveri et al., 2018; Chan et al., 2018), joint osteoarthritis (Neogi et al., 2013; Van Dijck et al., 2018), surgical planning and intervention (Zheng and Schumann, 2009; Cerveri et al., 2017; Mauler et al., 2017; Youn et al., 2017), and morphology-function relations (Fitzpatrick et al., 2011; Rao et al., 2013; Baka et al., 2014; Peloquin et al., 2014; Smoger et al., 2015; Hollenbeck et al., 2018; Cerveri et al., 2019b; Clouthier et al., 2019). There have, however, been few studies attempting to extensively investigate the relationship between morphological features and the degree of deformity of the tibio-femoral joint affecting the mechanical stability of the knee. This lack is probably due to the difficulty of considering the geometry of multiple bony structures and their relative position and to the complexity of building statistical models of pathological bones affected by severe deformations. In Rao et al., the authors elucidated the relationships between MoV and the relative alignment of the knee structures by means of an SSM built using magnetic resonance imaging of 20 knees (Rao et al., 2013). Interestingly, they reported that some mechanical features of the tibia (anterior-posterior alignment and varus-valgus angle) and the femur (internal-external rotation) were encoded by specific MoVs. However, the tibio-femoral 3D misalignment was not explicitly encoded in the MoVs. Smoger et al. proposed to link the knee articular geometry and kinematics using an SSM built on 20 cadaveric specimens considered normal from a clinical point of view. Joint kinematic data of knee flexion/extension, captured by Kansas knee simulator, were used to compare experimental angular variables to the one simulated by the SSM (Smoger et al., 2015). Correlations between specific shapes in the knee and tibio-femoral alignment were reported. However, SSM parameter variations were not general enough to produce sufficient pathological alteration and bone deformations. In Clouthier et al., the authors studied the correlation between SSM parameters and the biomechanical factors of the knee using a statistical model built on 14 asymptomatic knees composed of distal femur, patella, and proximal tibia (Clouthier et al., 2019). SSMs were used to generate a number of morphological configurations of the bones, and each one was embedded into a lower-extremity musculo-skeletal model to evaluate the corresponding knee mechanics during a simulated gait cycle. The authors examined changes in knee mechanics (both bone kinematics and contact forces) as a function of the specific SSM realization. However, the SSM construction and experimental tests were performed on healthy subjects, so that SSM parameter variations did not generate extensive pathological conditions. For example, changes in the frontal plane affected the mechanical alignment by at most ±3°, which is considered the normal range for frontal stability of the knee. Based on such literature and capitalizing on our previous works (Cerveri et al., 2017, 2018, 2019a,b), in this paper, an SSM of pathological bony shapes in the knee is proposed to investigate the correlation between MoVs and the mechanical deformity of tibia and femur, assumed to induce kinematic instability. The statistical shape model of the tibia-femur bone complex was built using 99 pathological cases. The deformity degree was described in terms of 3D tibio-femoral alignment (Figure 1), considering the HKA (α), FVV (β), TVV (γ), IER (θ), and TS (ω) angles. For each angular variable, a clinical range from the literature, representing average physiological conditions, was selected to define the boundary between stability and instability. For each knee joint, the MoV weights were computed and their relation with each angular variable investigated. Discriminant analysis and logistic regression models (Wang, 2014) were adopted to systematically study the relations between observations (stability/instability classes as a binomial variable) and MoV weights (covariates). In the light of these premises and leveraging the main hypothesis of relationship between shape and function, the proposed work aims at linking specific MoVs in the SSM to the parameters describing the tibio-femoral alignment. This can have an impact in the biomechanical and orthopedic domains, as it opens up the opportunity to predict knee instability by analyzing the femoral and tibial morphology in terms of MoVs expressed by the SSM without the need for direct landmark identification and analysis.

## 2. Materials and Methods

### 2.1. Patient Data

Digital bony shapes of distal femur and proximal tibia were extracted from a retrospective dataset of 100 patients (70 males and 30 females) provided in anonymized form by Medacta company (Medacta International SA, Castel S. Pietro, CH), including planning CT scans (acquired in a supine position for all patients) and reconstructed bony 3D surfaces (Cerveri et al., 2017, 2018). The patients, aged 67 ± 10 years, reported localized knee pain associated with mechanical knee instability at staging time. Diagnostic imaging confirmed different degrees of cartilage defects, femoral osteophytes, and shape abnormalities, mainly at the condylar regions of the distal femur and at the tibial plateau. All patients underwent knee resurfacing or knee replacement surgery between 2014 and 2016. For surgical planning purposes, the image acquisition protocol included computed tomographic (CT) scans of the knee, hip, and ankle regions. Each CT scan consisted of about 520 slices with an image resolution of 512 × 512 pixel and a voxel size of 0.48 × 0.48 × 0.5 mm. Expert radiological operators manually performed the image segmentation of the osseous portion of the proximal and distal femur as well as of the proximal and distal tibia using Mimics software (Materialize, Belgium). For each labeled CT volume, the 3D surface meshes, composed of vertices and triangular faces, were reconstructed automatically. For all the patients, HKA, FVV, IER, TVV, and TS were computed pre-operatively, exploiting landmarks manually detected on the surfaces. For SSM construction, distal femur and proximal tibia surfaces only were taken into account. As a function of the particular centering of the knee joint in the CT scan, the distal femur was segmented up to 2–4 cm away from the frontal notch of the trochlear region along the femur shaft. Similarly, the length of the proximal tibia shaft was variable across the set in a range of about 2–3 cm. Among the 100 cases, one was excluded from the set because of the presence of a fixation screw on the femoral shaft due to a previous intervention. All the valid surfaces underwent pre-processing by means of smoothing and sub-sampling starting from about 60,000 vertices, for both distal femur and proximal tibia samples, down to 10,000 vertices. Left surfaces were mirrored in the medio-lateral direction to obtain equivalent right surfaces for the construction of the right distal femur and proximal tibia SSM. The surface scale and the shaft lengths were not normalized. This is because, first, the normalization of a bundle of two shapes (femur and tibia) would have affected the relative size in between. Preserving the relative size of the two shapes in general increases the generality of the SSM (Pedoia et al., 2015). Second, the normalization would also affect the difference in the femur/tibial shaft lengths. The shaft length can be relevant for the bending in both sagittal and frontal planes.

### 2.2. Statistical Shape Model

In order to construct the SSM embedding femur and tibia shapes, the methodology extensively described in previous papers of our group was adopted, which is based on a pair-wise matching technique (Cerveri et al., 2017, 2018, 2019a). This technique rests on the manual selection of a reference geometry for aligning all the surfaces in the training dataset and computing robust point correspondences. In the present custom implementation, first, the two reference geometries (distal femur and proximal tibia) were randomly selected within a subset of surfaces featuring only small bone deformations. Second, they were meshed and smoothed to obtain average edge lengths of 1.5 mm, resulting in triangular surfaces containing about 6,000 nodes each. This number of vertices is similar to the number of surface nodes used in previous works in the literature (Zheng and Schumann, 2009; Subburaj et al., 2010; Zhang et al., 2014). Each pair of surfaces in the overall set of 99 samples (distal femur and tibia) was rigidly registered to the reference tibio-femoral shape so that the relative position and the joint space between the two surfaces were preserved without requiring additional constraints. The deformable registration, based on a coherent point drift algorithm (Myronenko and Song, 2010), required to determine the point correspondences was, conversely, performed separately for femur and tibia to ensure consistency of the deformation field. A robust algorithm for determining one-to-one point correspondences (Cerveri et al., 2019a) was adopted in this work. The number of correspondences was determined by the number of vertices of the reference shape. After computing the mean model $\bar{m}$ from point correspondences, the covariance matrix, obtained by stacking the femur and tibia distance data from the mean model, underwent principal component analysis, providing 98 independent MoVs. Each MoV was represented by the eigenvector *v*_*i*_ and the corresponding eigenvalue σ_*i*_. The percentage amount of morphological variation encoded by the *j*th MoV, termed explained variance (*EV*), was computed as:

(1) $$\begin{array}{l} EV_{j}=\frac{\sigma_{j}^{2}}{\sum_{i=1}^{M-1}\sigma_{i}^{2}} \end{array}$$

where *M* is the number of samples in the dataset. The effect of each MoV was expressed numerically by one weight λ that modulates the corresponding eigenvalue, where a value of 0 denotes the mean shape, and negative and positive values represent the deviance from this mean in either direction. Accordingly, the SSM-based surface reconstruction, named morphing, was defined by the following equation:

(2) $$\begin{array}{l} Š=\bar{m}+\overset{M-1}{\underset{i=1}{\sum }}\lambda_{i}\sigma_{i}v_{i} \end{array}$$

where the reconstructed surface Š is obtained by summing up the mean model $\bar{m}$ with the series of all MoVs. For each surface, the weights were computed by projecting the shape pair (distal femur and proximal tibia) on the SSM by means of the scalar product (Cerveri et al., 2018). We retained enough MoVs to describe 95% of the overall shape variation, expressed by the cumulative *EV*, in the study population. The reconstructed surfaces were compared with the corresponding samples in the set by means of the surface distance error distribution (mean ± SD) using the Hausdorff distance.

### 2.3. Modeling Tibio-Femoral Alignment by SSM Parameters

**Figure 2:**
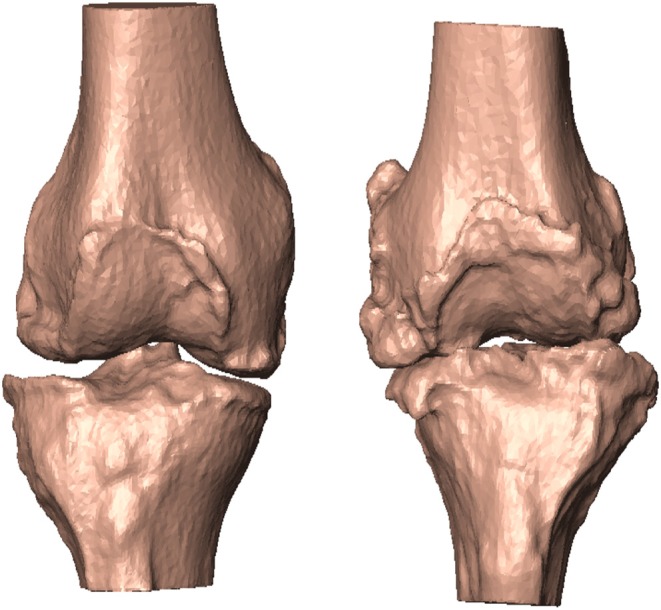
According to the five angular quantities, patient #20 featured no alignment deformation **(left)**. Patient #29, in contrast, featured mechanical instability in all but one (IER) angular quantities **(right)**.

In order to study the association between the MoVs and the condition of knee misalignment, the following normality ranges of the clinical variables were first considered: HKA: 0°±3, FVV: −6°±2 (physiological valgum), IER: ±5°, TVV: ±5°, TS: 7°±4 (Salenius and Vankka, 1975; Iranpour-Boroujeni et al., 2014; Driban et al., 2016; Schatka et al., 2018). According to each clinical variable, the 99 cases were separated into two classes, stable and unstable (Table 1). Note that the same instance may be considered stable according to one clinical parameter while being unstable according to another. As an example, two very different cases are depicted in Figure 2, the first lying within physiological ranges according to all the five angular quantities and the second featuring mechanical instability according to all but one (IER) angular quantities. We adopted two different data processing techniques, namely the discriminant analysis (DA) and the logistic regression (LR) classifiers. Both linear (LDA) and quadratic (QDA) discriminant analysis techniques were applied for the classification and the detection of low-dimensional sets of MoVs able to separate the stability from the instability condition. The accuracy (AC), sensitivity (SE), and specificity (SP) of the classification were computed for each dependent variable (clinical quantities) with respect to the explanatory variables (SSM parameters) using the leave-one-out (LOO) cross-validation technique. LOO classification based on LR was computed, and the statistical association (*p* < 5%) between SSM parameters and the clinical quantities was determined. In order to further understand the contribution of each MoV in discriminating between stable and unstable conditions, the distributions of relevant MoVs (significant according to the previous analysis) were compared in the two conditions using a Wilcoxon signed-rank test (*p* = 0.05). Moreover, the correlations between the instability grade and each MoV were investigated. In other words, it was assumed that instability increased as the clinical parameter values drifted away from the reference physiological range and looked for a correspondence in MoV weight variations. Since both angle increases and angle decreases from the normal values relate to instability, a variable change was implemented, introducing a corrected version $\hat{X}=|X-\bar{X}|$ of the clinical parameters by computing the absolute value of the difference between the parameter itself and its physiological average value, where *X* is a generic clinical parameter, $\bar{X}$ is its average value (in physiological cases), and $\hat{X}$ is its corrected form. The correlation between the MoVs and the corrected parameters was assessed by means of the Spearman coefficient (*p* = 0.05).

**Table 1:** Stability/Instability class definitions according to the thresholds for the five clinical variables.

**Condition**	**HKA**	**FVV**	**IER**	**TVV**	**TS**
Stability	28	25	84	79	70
Instability	71	74	15	20	29

## 3. Results

### 3.1. Relation of MoVs to Bone Morphological Variability

**Figure 3:**
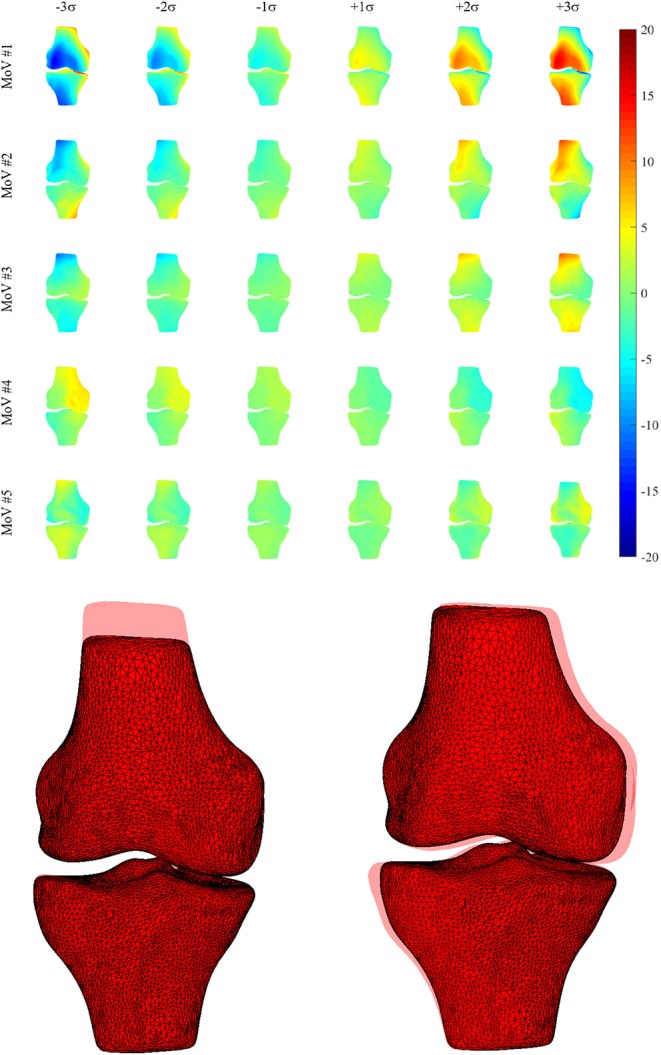
**(Upper)** Morphological deviations (mm) from the mean model (frontal view) for MoVs from #1 to #5. **(Lower)** Explicit morphology in two SSM parameter sets (MoV #3 -3σ, MoV #4 +3σ) with the overlaid mean model.

Thirty-four MoVs were sufficient to describe 95% of the shape variability. Quantitatively, the reconstruction error across the 99 surfaces was 1.38 ± 0.16 mm. Qualitatively, the first (*EV*_1_ = 36.4%) MoV primarily encoded the isotropic scale of the bone complex. MoV #2 (*EV*_2_ = 16.3%) represented the size and height of the shafts of the two bones, concurrently. It also represented the shaft bending, mainly in the frontal plane (see Table 2). MoV #3 (*EV*_3_ = 9.9%) modeled the elongation of the femoral shaft (λ_3_ > 0) and the shortening of the tibial shaft (λ_3_ < 0). MoV #4 (*EV*_4_ = 5.6%) encoded the enlargement of the tibial plateau and the relative translation between the two shapes in the mediolateral direction. MoV #5 (*EV*_5_ = 3.6%) represented variations of the axial section of the femoral and tibial shafts and the relative bending between the two bones, in both the frontal and sagittal planes (Table 2 and Figure 3). Positive values of the weight also encoded hypoplasia effects of the anterior facet of the medial condyle. MoV #6 (*EV*_6_ = 2.6%) described the concurrent modification of the anterior-posterior size of the femoral condylar region and that of the tibial plateau. Positive values of MoV #6 represented abnormal flatness in the trochlear region of the femur. This is unlike MoV #7 (*EV*_7_ = 2.1%), which modeled the tibial and femoral medio-lateral size, with positive values representing bone shrinkage. MoV #8 (*EV*_8_ = 1.9%) again represented the bending in the frontal plane between the two bones; however, the bending represented by MoV #5 was associated with a concurrent shrinkage/enlargement of the two shaft diameters, which was not encoded by MoV #8. MoV #9 (*EV*_9_ = 1.6%) modeled the medio-lateral shrinkage of the tibial plateau, with a concurrent anterio-posterior enlargement of the condylar region of the femur, up to pathological flattening. MoV #10 (*EV*_1_0 = 1.6%) represented tibial inclination in both the frontal and sagittal planes. MoV #11 (*EV*_1_1 = 1.3%) mainly represented tibia inclination on the sagittal plane, with a concurrent bending of the femur on the same plane. MoV #12 (*EV*_1_2 = 1.1%) modeled a slight femoral bending on the frontal plane. MoV #13 (*EV*_1_3 = 1.1%) modeled the relative bending between the two bones in the sagittal plane. The remaining MoVs represented small and spread morphological variations and was not straightforward to visually relate any to specific local features (see Supplementary Materials).

**Table 2:** Morphological variability of femur (F) and tibia (T) and relative alignments mapped onto the MoVs from 2 to 9.

**Bone variability**	**MoV #2**	**MoV #3**	**MoV #4**	**MoV #5**	**MoV #6**	**MoV #7**	**MoV #8**	**MoV #9**
F shaft elongation	o	o						
F shaft diameter	o			o				
F shaft bending	o			o			o	
F condylar AP size					o			o
F condylar ML size						o		
T shaft elongation	o	o						
T shaft diameter	o			o				
T shaft bending	o			o			o	
T plateau AP size			o		o			
T plateau ML size						o		o

### 3.2. MoV Performance in Instability Modeling

#### 3.2.1. Discriminant Analysis

Table 3 shows the classification performances (sensitivity, specificity, and accuracy) obtained using the LOO procedure for both the linear discriminant analysis and quadratic discriminant analysis (34 MoVs were employed in the task). Despite the fact that the LDA accuracy ranged between 0.7 and 0.84, the respective values of sensitivity and specificity were highly different: in two cases (IER and TS), the sensitivity was lower than 0.4 (specificity > 0.8), while, conversely, for HKA and FVV the sensitivity was as high as 0.85, while specificity reached 0.64 and 0.44, respectively. As far as QDA is concerned, higher accuracy levels (range: 0.87–1) corresponded instead with both high sensitivity (range: 0.79–1) and high specificity (range: 0.86–1). It is worth noticing that reducing the MoVs to the three or four most relevant as shown in Tables 4, 5, respectively, reduces the performance, again causing specificity/sensitivity mismatches. In fact, in both cases (Tables 3, 4), poor accuracy was found for TVV (0.4 and 0.55) and TS (0.44 and 0.48), while FVV (0.32 and 0.60) resulted in low specificity. Nevertheless, it has to be pointed out that in Table 5, using four MoVs, only one value fell slightly below the threshold of 0.5 (TS sensitivity: 0.48) suggesting that, even in this reduced form, QDA was able to outperform LDA. Interestingly, considering the four-MoV-based QDA, both MoVs #2 and #5 were representative of all of the clinical measures except for TVV (MoV #5 only).

**Table 3:** Classification test, exploiting LOO cross-validation, of linear vs. quadratic discriminant analysis using all SSM parameters.

	**LDA-AC**	**LDA-SE**	**LDA-SP**	**QDA-AC**	**QDA-SE**	**QDA-SP**
HKA	0.82	0.88	0.64	0.96	0.96	1
FVV	0.74	0.85	0.44	0.99	0.99	1
IER	0.80	0.33	0.89	0.88	1	0.86
TVV	0.84	0.55	0.92	1	1	1
TS	0.70	0.37	0.82	0.87	0.79	0.91

**Table 4:** The three most representative SSM parameters for quadratic discriminant analysis.

	**MoVs**	**QDA-AC**	**QDA-SE**	**QDA-SP**
HKA	2, 5, 13	0.83	0.88	0.71
FVV	5, 14, 17	0.79	0.95	0.32
IER	2, 9, 14	0.92	0.53	1
TVV	5, 12, 15	0.87	0.40	1
TS	3, 13, 18	0.81	0.44	0.97

**Table 5:** The four most representative SSM parameters for quadratic discriminant analysis.

	**Param**	**QDA-AC**	**QDA-SE**	**QDA-SP**
HKA	2, 5, 11, 17	0.88	0.93	0.75
FVV	2, 5, 10, 17	0.83	0.91	0.60
IER	2, 8, 9, 12	0.95	0.66	1
TVV	5, 12, 14, 15	0.88	0.55	0.97
TS	2, 5, 8, 11	0.84	0.48	0.99

#### 3.2.2. Logistic Regression

**Figure 4:**
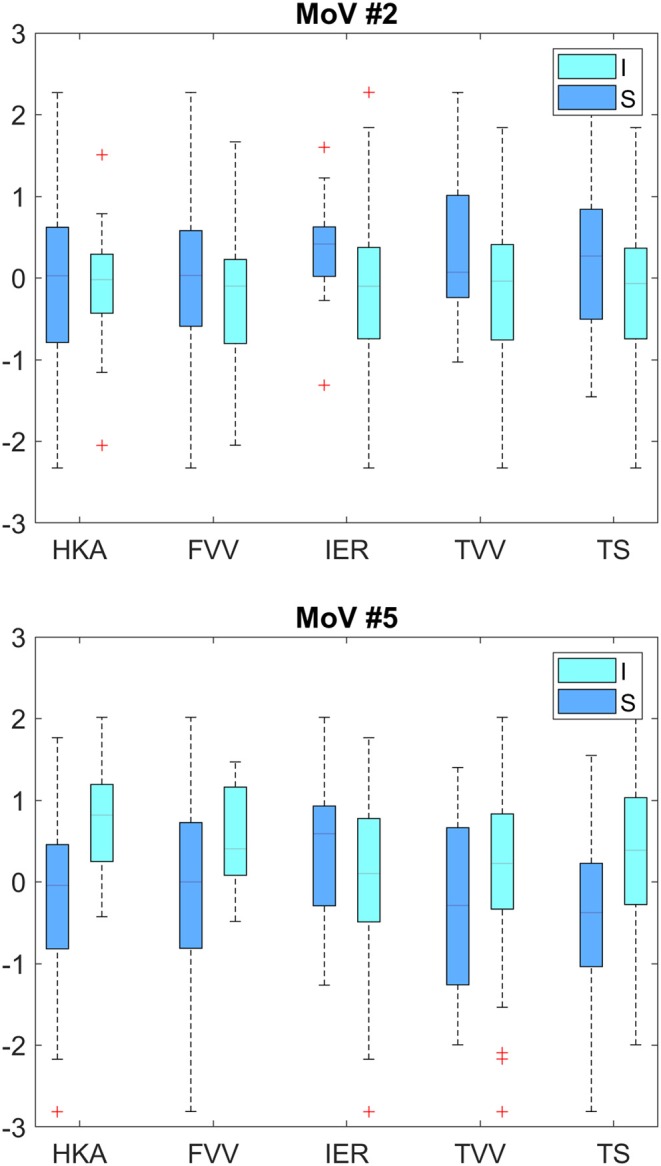
Box plots for MoV #2 and #5 distributions across mechanically stable and unstable cases. Upper plot—HKA (*p* = 0.90), VVF (*p* = 0.08), IER (*p* = 0.02), VVT (*p* = 0.08), and TS (*p* = 0.06). Lower plot—HKA (*p* = 0.0005), VVF (*p* = 0.01), IER (*p* = 0.13), VVT (*p* = 0.09), and TS (*p* = 0.002). I, Instability; S, Stability.

**Figure 5:**
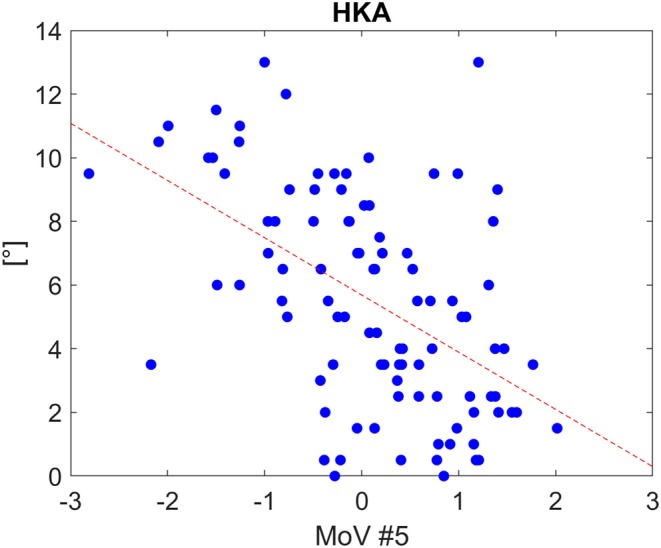
Scatter plot for HKA distribution as a function of MoV #5 range, along with the linear fitting (Spearman correlation coefficients: −0.52 with p<1e-07). The tendency line is depicted (red dashed line).

Classification results (AC, SE, SP) with LOO cross-validation for HKA, FVV, IER, TVV, and TS were (0.79, 0.88, 0.57), (0.83, 0.93, 0.56), (0.84, 0.13, 0.97), (0.83, 0.75, 0.97), and (0.75, 0.31, 0.94), respectively. The statistical analysis provided significance (*p* < 0.05) in HKA for MoVs #5, #7, and #18, FVV for MoV #5, IER for MoV#2, TVV for MoVs #11, #14, #16, and #17, and TS for MoV #5 (Table 6). Nicely, MoVs #2 and MoV #5 were found to be largely representative of the logistic modeling, in agreement with the DA results. For these two MoVs, the box plots were reported in order to highlight the distribution differences across mechanically stable and unstable cases for each clinical parameter (Figure 4). As far as MoV #2 is concerned, stability and instability were significantly different in IER distributions (*p* = 0.02). As far as MoV #5 is concerned, both HKA (*p* = 0.0005) and TS (*p* = 0.002) resulted in significant differences. As far as the correlation analysis is concerned, MoV #5 showed significant correlation with $\hat{HKA}$ (c = −0.52, *p* < 10^−7^), $\hat{FVV}$ (c = −0.26, *p* < 0.01), and $\hat{TS}$ (c = −0.23, *p* < 0.03). Likewise, MoV #7 showed significant correlation with $\hat{HKA}$ (c = −0.27, *p* < 0.008) and $\hat{FVV}$ (c = −0.32, *p* < 0.002), while MoV #6 resulted in significant correlation only in the case of $\hat{FVV}$ (c = 0.25, *p* < 0.02) (see Table 7). A scatter plot showing $\hat{HKA}$ against MoV #5 was reported in Figure 5.

**Table 6:** SSM parameters that were statistically significant for the logistic regression.

	**MoV/p value**
HKA	5 (*p* = 0.0001), 7 (*p* = 0.03), 17 (*p* = 0.02), 18 (*p* = 0.03)
FVV	5 (*p* = 0.001), 10 (*p* = 0.01), 17 (*p* = 0.01)
IER	2 (*p* = 0.02)
TVV	11 (*p* = 0.008), 14 (*p* = 0.01), 16 (*p* = 0.01), 17 (*p* = 0.03)
TS	5 (*p* = 0.02)

**Table 7:** Correlation intensity (Spearman coefficient c) and significance (*p*-value) between relevant MoVs and clinical parameters defining knee instability.

**MoV**	**HKA**		**FVV**		**IER**		**TVV**		**TS**	
	**c**	**p**	**c**	**p**	**c**	**p**	**c**	**p**	**c**	**p**
#1	−0.14	0.17	−0.09	0.36	0.06	0.53	−0.17	0.09	0.15	0.15
#2	0.11	0.26	0.10	0.35	0.18	0.07	0.09	0.40	0.03	0.74
#3	0.14	0.18	0.02	0.84	−0.07	0.47	−0.04	0.68	0.17	0.09
#4	0.12	0.22	0.17	0.10	0.09	0.35	0.05	0.59	0.11	0.26
#5	−0.52	10^−8^	−0.26	0.01	0.08	0.44	−0.17	0.10	−0.23	0.02
#6	0.14	0.16	0.25	0.01	0.03	0.78	0.15	0.12	0.07	0.49
#7	−0.27	0.01	−0.32	0.001	0.05	0.63	−0.11	0.27	0.08	0.43
#8	−0.11	0.28	0.01	0.92	0.16	0.12	10^−4^	0.99	−0.10	0.32
#9	0.03	0.78	−0.05	0.65	0.19	0.05	0.01	0.90	0.07	0.47
#10	−0.05	0.59	−0.10	0.34	−0.02	0.82	−0.05	0.65	−0.02	0.83

*MoVs #5 and #7 feature significant correlation results*.

## 4. Discussion and Conclusions

### 4.1. Findings, Limitations, and Possible Developments

Computational approaches to study the correlation between morphological features and functional or pathological conditions of bony surfaces using SSM have been emerging in the literature, with impacts in biomechanics, especially for kinematic and dynamic analysis (Rao et al., 2013; Smoger et al., 2015; Nolte et al., 2016; Zhang et al., 2016; Hollenbeck et al., 2018; Clouthier et al., 2019), and clinics, especially for diagnostic and surgical interests (Neogi et al., 2013; Peloquin et al., 2014; Mutsvangwa et al., 2015; Cerveri et al., 2018). In particular, three studies addressed the relation between SSM parameters and knee kinematics by focusing on the link between the morphological variability of the bones and tibio-femoral alignment modifications (Rao et al., 2013; Smoger et al., 2015; Clouthier et al., 2019). The main issue of such studies was that the relationships between shape and alignment were simulated by systematically perturbing MoVs, reaching up to 95% variation with respect to the mean model. However, SSMs were computed using a very small group of asymptomatic cases. Therefore, pathological conditions were not explicitly encoded in the MoVs, leading to the simulation of mechanical axis misalignment within normality ranges. This hindered the model's ability to extrapolate non-physiological conditions of the knee. Conversely, in our work, the SSM analysis was addressed by considering a population of 99 knee cases with different morphological and mechanical anomalies at:

- the distal femur, namely condylar enlargement, osteophytes, notch narrowing, trochlear flattening;
- the proximal tibia, namely plateau enlargement, osteophytes, smoothing of the intercondylar eminence, flattening of the tibial tuberosity.

Moreover, for each case, the tibio-femoral alignment of the knee was measured not only in the frontal plane (HKA, TVV, and FVV) but also in the sagittal (TS) and axial (IER) planes. We built an SSM using the two bone sets (distal femur and proximal tibia), computed the MoV weights for each case, tested both DA and LR classifiers of stability/instability as a function of MoV weights, and analyzed the relevance of each MoV for joint instability. The major findings of the work can be summarized as:

- the computed SSM was representative of the surface set, demonstrated by the very low reconstruction error;
- the physiological and pathological variations of the knee morphology found in the surface dataset were consistently encoded by 34 MoVs (EV > 0.95);
- together, MoV #2 (height/size of femoral/tibial shafts) and MoV #5 (femoral/tibial bending in the frontal/sagittal plane) were the most relevant MoVs, representing a total of about 20% of the variation among SSMs;
- MoV classification results were largely in agreement with morphological features determining tibio-femoral instability (compare Table 2 with Tables 4, 5);
- QDA outperformed LDA in classifying unstable versus stable cases with high accuracy for all the five clinical parameters;
- despite the fact that the LR-based classification provided lower-accuracy results, statistically significant MoVs were in agreement with QDA.

Synthetically, these findings suggest that the computed SSM can be exploited for assessing whether a knee lies in a pathological condition according to the more traditional clinical parameters, namely HKA, FVV, IER, TVV, and TS, without the need for landmark selection, just fitting the SSM to the shape of interest. In more detail, the SSM decomposition showed that the first 13 were sufficient to describe 85% of the explained variance, demonstrating the SSM's ability to model large morphological variability in a very compact way. MoVs of the SSM were also related to tibio-femoral alignment and knee instability according to the five clinical parameters considered. This was confirmed by the classification performance, because four MoVs (see Table 5) were able to ensure more than 80% of accuracy in the quadratic discriminant analysis. Again, this makes SSM a prospective candidate tool for distinguishing stable and unstable knee conditions by analyzing the surfaces only, without the manual definition of rotation centers and mechanical axes.

An in-depth analysis of the classification performances showed that the LDA model was under-fitting. The size imbalance of the two classes (see Table 1) further contributed to bias the results. This was evident (see Table 3), for example, when considering the HKA (featuring only 28 stable cases with respect to 71 unstable cases) and IER (only 15 unstable conditions). Conversely, QDA appeared to be more robust to dataset imbalance, showing both higher sensitivity and specificity than LDA. The LR analysis highlighted a couple of MoVs relevant for discriminating between stability and instability, namely MoVs #2 and #5, representative of all the clinical measures. These two specific MoVs were found to be significant to discriminate between stability and instability. Specifically, MoV #2 mainly encoded the elongation and partially encoded the bending of the two shafts. This is in agreement with the relation with the variation of the two mechanical axes and, by consequence, with their relative inclination. This can therefore be related to the joint mechanical alignment, especially in the frontal plane. MoV #5, encoding the relative bending of the two bones in the frontal plane (see Table 2), was confirmed to be related to the HKA, FVV, and TVV angles, which describe the tibio-femoral stability in the frontal plane. As confirmed by the classification results, this MoV was able to discriminate between stability and instability. In synthesis, while the effect of these two MoVs could not be predicted a priori, the morphological aspects encoded by both of them could reasonably be considered to be related to the tibio-femoral alignment. It has to be pointed out, however, that differences in the bone shaft heights encoded in MoV #2 were caused by different ranges of interest in CT scans and could not be ascribed to morphological variability. However, it is reasonable to assume that the frontal bending and lateral inclination of the shaft are morphological features relevant for the overall tibio-femoral alignment. Therefore, MoV #2 was not discarded, a choice that was justified a-posteriori, considering that it was relevant for the classification.

One shortcoming of our work is the inclusion of just the femoral and tibial surfaces, neglecting the patellar region and the cartilages. Nonetheless, this choice was motivated by previous literature findings showing that increasing the number of geometries to be included in the SSM can easily lead to difficulty in identifying specific correspondences between MoVs and morphological features. For instance, Fitzpatrick et al. used 26 healthy subjects to develop an SSM of the patello-femoral joint, reporting that the main variability of the patella articular curvature and sulcus groove was actually spread across many different MoVs (Fitzpatrick et al., 2011). As a matter of fact, our approach allowed us to identify correlations between specific MoVs and clinical parameters of the alignment between femur and tibia. In this paper, we did not investigate how a different reference shape selection would have affected the reconstruction and the classification results. The reference shape was selected randomly from a subset of surfaces little-affected by deformities. This was in agreement with the results reported in a recent paper with similar acquisition techniques, where the random selection of the SSM also had little effect (Cerveri et al., 2019a). As far as data acquisition is concerned, all the patients were lying supine during CT acquisition and images were acquired using the same protocol. As regards data processing, the images were segmented by different expert radiological operators. Each scan was segmented by one operator, so we did not have any information about variability in segmentation. Similarly, the landmark detection and the angle computation were performed by one expert orthopedic surgeon. As the scans were all at sub-millimeter resolution, the bony segmented profiles were affected by such uncertainty, which was present in the final surface reconstruction. The surface sub-sampling lowered, on average, the surface quality by <2% (root-mean-squared-distance: <1 mm) with respect to the original surfaces. The SSM reconstruction error was, on average, lower than 1.5 mm, reasonably localized in the region affected by the largest pathological deformations. Actually, we focused on the overall SSM reconstruction ability without taking local errors into account. The analysis of the reconstruction quality in critical regions heavily affected by deformities (e.g., the presence of large osteophytes) could have provided further information about the specificity and generality of the SSM model. However, this analysis would have required a greater effort by the expert operators to manually detect and classify the regions with severe deformations, which is a very time-consuming task beyond the scope of the present work. Conversely, we aimed at relating MoV weights to angular stability determined by the five clinical indices. It is reasonable to assume that the local reconstruction errors should affect the overall knee joint alignment less, which should be mainly determined by the overall bony shape. Nevertheless, analysis of how local reconstruction errors could affect the relative 3D rotation may be carried out in a future study by means of a sensitivity analysis. Finally, the SSM model could be used to study the development of stress and strain in the knee due to applied loads as a function of surface geometry changes. This would require that a finite element description be integrated into the SSM to perform the computations, which could be used to predict the outcome of surgery, taking into account patient-specific variability. Moreover, in order to analyze the effects of the relative tibio-femoral position and orientation on gait motion patterns, the SSM could be combined with the angular trajectories reconstructed using surface markers acquired by means of an opto-electronic motion capture system. For instance, simulations could be useful for evaluating how gait cycle parameters (e.g., gait cadence, step length, etc.) would be affected. Likewise, the SSM could help to study, in knee surgical planning, how the tibio-femoral spacing would change the flexion-extension patterns of knee.

### 4.2. Literature Comparison

Rao et al. developed an SSM of the femur, tibia, and patella of 20 cadavers, considering different alignments obtained by using a mechanical simulator applied to the specimens (Rao et al., 2013). About 95% of the variability was captured by just 15 MoVs. Fitzpatrick et al. used 26 healthy subjects to develop an SSM model of the patello-femoral joint (Fitzpatrick et al., 2011). Similarly, 15 MoVs were sufficient to capture about 97% of the morphological variability. Fourteen asymptomatic patients scanned by MRI were used in Clouthier et al. to build an SSM of the knee that was able to represent 70% of the variability by means of 6 MoVs only (Clouthier et al., 2019). In our work, we used a wide dataset of pathological knees featuring large anomalies at both femoral and tibial sites. As a consequence, the greater number of MoVs needed to represent most of the variability (34 MoVs accounting for 95% EV) was to be expected. This corroborates the consideration that morphological abnormalities cannot be simply extrapolated by an SSM built on normal joints. In other words, femoral and tibial deformities cannot be represented just by enlarging the weight range of the MoVs (e.g., ±3, ±4, ±5 SD, etc), but, rather, there is a need to encode such information in new MoVs entirely. This is also in agreement with the limitations acknowledged in the previous literature (Fitzpatrick et al., 2011; Rao et al., 2013; Smoger et al., 2015; Clouthier et al., 2019). For instance, Hollenbeck et al. reported that a maximum range of ±2 SD was allowed in their lumbar spine SMM in order to avoid unrealistic deformations (Hollenbeck et al., 2018). As far as the relation between MoVs and kinematics is concerned, Smoger et al. reported that their third MoV described differences in the internal-external relative rotation between femur and tibia (Smoger et al., 2015); this was nicely in agreement with our results for MoV #5. Internal−external alignment of the patellofemoral joint was described by the second mode in Rao et al. (2013). Interestingly, they also found tibial internal–external rotation and tibial varus-valgus variations encoded by the third and fourth MoVs, respectively. However, femoral alignments were not modeled by their SSM. In Pedoia et al., the authors developed distal femur and proximal tibia SSMs, avoiding the normalization of the samples (Pedoia et al., 2015). They reported that the first mode was related to the size for both SSMs, as in our case. For the femur, modes #2 and #3 were related to the relative distance between the condyles and the condylar width and height, respectively. In our model, these features were mainly encoded by MoV #6 and #7. As far as the tibia was concerned, modes #2 and #3 were related to the medial posterior curvature of the tibial plateau and the elevation of the anteromedial tibial plateau, respectively. In our model, these morphological features were spread across MoVs #4, #6, and #7. These differences were expected because we dealt with a unique SSM for the tibio-femoral joint. Our method may provide insights regarding concurrent morphological deformations in the two bones.

## 5. Conclusion

**Figure 6:**
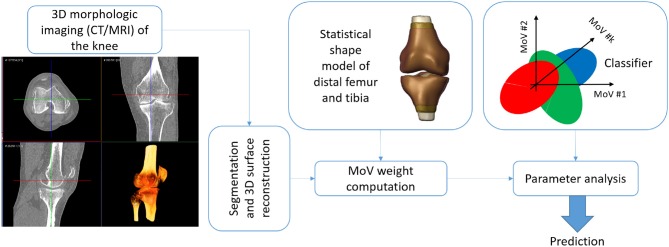
The application workflow.

The SSM approach was proven to consistently represent both morphological anomalies and alignment deviation in the knee bones by means of few representative MoVs. In the light of such results, the proposed SSM met the objectives of providing an alternative to manual definition of bone landmarks to assess pathological conditions related to knee instability. The SSM could be exploited in a decision support system that predicts the potential instability of the joint by processing the knee scan without requiring images of other body regions (e.g., hip and ankle) and with no need for manual landmark identification. This toolbox could thus generate an automatic report with a diagnosis of stable or unstable according to each clinical variable of the five indexes considered. A potential application workflow would rest on: (1) the bone segmentation in the knee scan; (2) the surface reconstruction; (3) the weight computation by the SSM; (4) the prediction of the instability based on the five different clinical factors of alignment by means of a classifier (e.g., discriminant analysis) (Figure 6). Another possible exploitation of the proposed SSM approach is the simulation of the effects of different morphological conditions (achieved by varying MoV weights) on movement analysis of the knee, as suggested by Smoger et al. (2015) and Clouthier et al. (2019), studies that both proposed SSMs built on healthy subjects. An SSM including large pathological variability, such as the one developed in this work, opens up the opportunity to study the effect of a specific misalignment of the femur and tibia on the simulated motion pattern and, consequently, the resulting load distribution affecting cartilage wear.

## Data Availability Statement

The datasets generated for this study are available on request to the corresponding author.

## Ethics Statement

The study involved retrospective anonymized digital data provided by Medacta International SA (CH) in accordance with the institutional ethical committee.

## Author Contributions

PC performed SW implementation, data processing and analysis, and manuscript writing. AB contributed to data analysis and manuscript writing. AM contributed to biomechanical and clinical data interpretation, and manuscript commenting.

## Conflict of Interest

The authors declare that the research was conducted in the absence of any commercial or financial relationships that could be construed as a potential conflict of interest. The reviewer AM declared a shared affiliation, with no collaboration, with the authors, PC and AB, to the handling editor at the time of the review.
